# Host‐induced silencing of essential genes in *Puccinia triticina* through transgenic expression of RNAi sequences reduces severity of leaf rust infection in wheat

**DOI:** 10.1111/pbi.12845

**Published:** 2017-12-15

**Authors:** Vinay Panwar, Mark Jordan, Brent McCallum, Guus Bakkeren

**Affiliations:** ^1^ Agriculture and Agri‐Food Canada Morden Research and Development Centre Morden MB Canada; ^2^ Agriculture and Agri‐Food Canada Summerland Research and Development Centre Summerland BC Canada

**Keywords:** *Puccinia triticina*, wheat leaf rust, host‐induced gene silencing, RNAi, transgenic wheat, rust resistance

## Abstract

Leaf rust, caused by the pathogenic fungus *Puccinia triticina* (*Pt*), is one of the most serious biotic threats to sustainable wheat production worldwide. This obligate biotrophic pathogen is prevalent worldwide and is known for rapid adaptive evolution to overcome resistant wheat varieties. Novel disease control approaches are therefore required to minimize the yield losses caused by *Pt*. Having shown previously the potential of host‐delivered RNA interference (HD‐RNAi) in functional screening of *Pt* genes involved in pathogenesis, we here evaluated the use of this technology in transgenic wheat plants as a method to achieve protection against wheat leaf rust (WLR) infection. Stable expression of hairpin RNAi constructs with sequence homology to *Pt* MAP‐kinase (*PtMAPK1*) or a cyclophilin (*PtCYC1*) encoding gene in susceptible wheat plants showed efficient silencing of the corresponding genes in the interacting fungus resulting in disease resistance throughout the T_2_ generation. Inhibition of *Pt* proliferation in transgenic lines by *in planta*‐induced RNAi was associated with significant reduction in target fungal transcript abundance and reduced fungal biomass accumulation in highly resistant plants. Disease protection was correlated with the presence of siRNA molecules specific to targeted fungal genes in the transgenic lines harbouring the complementary HD‐RNAi construct. This work demonstrates that generating transgenic wheat plants expressing RNAi‐inducing transgenes to silence essential genes in rust fungi can provide effective disease resistance, thus opening an alternative way for developing rust‐resistant crops.

## Introduction

Rust fungi, belonging to the genus *Puccinia*, are among the most economically destructive biotrophic phytopathogenic fungi infecting cereal crops. Members of this genus pose a serious threat to global wheat production which is a major staple food for mankind in many parts of the world. Wheat is a host to three important rust pathogen species: *Puccinia graminis* f. sp. *tritici* (*Pgt*), *Puccinia striiformis* f. sp. *tritici* (*Pst*) and *Puccinia triticina* (*Pt*), which are the causal agents of stem rust, stripe rust and leaf rust, respectively (Bolton *et al*., 2008; Chen *et al*., 2014; Singh *et al*., 2011). Of the three rust diseases, wheat leaf rust (WLR, caused by *Pt*) is comparatively more prevalent occurring regularly wherever wheat is grown (Kolmer, 2005). Because of its adaptation to diverse climatic conditions and widespread occurrence, leaf rust results in greater total annual losses worldwide than stem and stripe rusts (Huerta‐Espino *et al*., 2011). Under severe epidemic conditions, *Pt* can inflict yield losses ranging from 10% to 70% (Herrera‐Foessel *et al*., 2006). Fungicides are usually applied for management of rust diseases, but financial costs and adverse environmental impacts are associated with chemical inputs. In addition, a major concern with excessive application of antimicrobial compounds is a high risk of reduced sensitivity or development of resistant pathogen populations.

Genetic resistance remains the most economical, effective and ecologically sustainable approach to minimize the extent of crop damage from leaf rust disease (Draz *et al*., 2015). Current disease control measures against *Pt* rely predominantly on breeding and development of resistant cultivars. So far, more than 70 leaf rust resistance (*R*) genes have been reported, the majority of which confers protection to a particular genetic variant of pathogen species mediated by gene‐for‐gene‐type interactions (Ellis *et al*., 2014; Flor, 1971; Singla *et al*., 2017). However, resistance alleles deployed into elite cultivars followed by monoculture farming instigate enormous selection pressures on rust pathogens. This often leads to new virulent races of the fungus, evolved to avoid recognition by these alleles and thereby rendering the resistant variety ineffective in a relatively short time (McCallum *et al*., 2016; Webb and Fellers, 2006). Further, finding and introgressing durable resistance genes from wild donor species without any undesirable trait drag remain a challenge to breeders. It is expected that rapid evolutionary dynamics of rust pathogens combined with anthropogenic climate changes will accelerate disease outbreaks in cultivated wheat worldwide (Helfer, 2014). Therefore, a continuous search for developing alternative biotechnological solutions is required to alleviate the negative impact of rust pathogens in wheat.

*Pt* is a highly specialized macrocyclic, heteroecious fungus having a complex life cycle involving up to five different spore stages on two taxonomically different host plants with species in Triticeae being the primary hosts (Bolton *et al*., 2008). The fungus reproduces on wheat by the formation of asexual dikaryotic urediniospores, and under favourable conditions, this phase can lead to multiple infection cycles thereby causing crop failure within weeks. *Pt* infection in wheat occurs via urediniospore germ tubes that differentiate to form appressoria over leaf stomata to penetrate into substomatal cavities. Subsequently, the plant tissue is colonized with an intercellular mycelium, breaching plant cell walls and invaginating the plasma membrane to form haustoria. This intimate connection surrounded by a zone of extrahaustorial matrix marks the basis of molecular crosstalk between the host and fungal cells (Garnica *et al*., 2014). Through haustoria, the fungus acquires nutrients from the host and there is evidence to suggest that these structures also play a crucial role in the delivery of virulence effectors to suppress host defence responses (Petre *et al*., 2015; Struck, 2015). The asexual cycle is completed within 7–10 days when sporogenous uredinia are formed containing thousands of infective propagules that are easily wind‐dispersed over long distances to establish new infections.

The availability of many cereal rust fungus genome sequences, along with the integration of comparative analyses, has contributed valuable insight into genome organization and annotation of genes involved in pathogenesis (Cantu *et al*., 2011; Cuomo *et al*., 2017; Duplessis *et al*., 2011; Wu *et al*., 2017; Zheng *et al*., 2013). However, attempts to translate this knowledge to decipher pathogen biology are hampered because these biotrophic fungi are not amenable to many of the traditional molecular genetic approaches, mainly due to difficulties in culturing these species outside of their host. In the postgenomics era, RNA interference (RNAi) has emerged as a powerful technique for *de novo* discovery of gene function. This technique, termed post‐transcriptional gene silencing (PTGS) in plants, involves the endonucleolytic cleavage of double‐stranded RNA (dsRNA) into small, ~20–23 nucleotide (nt)‐long, small interfering RNA (siRNA) duplexes by the Dicer‐like (DCL) enzymes which along with their associated proteins control the expression of genetic information (Baulcombe, 2004; Wilson and Doudna, 2013). An RNAi‐based approach referred to as host‐induced gene silencing (HIGS) or host‐delivered RNAi (HD‐RNAi) in which small RNAs are produced by the host plant to target parasite transcripts has provided a promising strategy for improving plant resistance against pathogens, including fungi and oomycetes (Andrade *et al*., 2015; Govindarajulu *et al*., 2015; Huang *et al*., 2006; Jahan *et al*., 2015; Koch and Kogel, 2014; Koch *et al*., 2013; Nowara *et al*., 2010; Panwar *et al*., 2013a, 2016; Song and Thomma, 2016; Yin *et al*., 2010). When targeted against crucial pathogenicity genes, HD‐RNAi can potentially be developed as a genetic method to curb pathogen virulence for pesticide‐free disease control in crop plants.

Recently, functional genomics assays based on HIGS methodology were developed for rust fungi, facilitating the characterization of some rust fungi genes required for virulence (Panwar *et al*., 2013a,b; Yin *et al*., 2014; Zhang *et al*., 2012). These studies harnessed transient gene silencing approaches, either delivering the HD‐RNAi constructs through *Barley stripe mosaic virus* (BSMV) or *Agrobacterium tumefaciens*‐mediated transfer. Applying these methods, we reported that *Pt* Map‐kinase (*PtMAPK1*), cyclophilin (*PtCYC1*) and calcineurin B (*PtCNB1*) encoding genes were required for full pathogenicity on the wheat host (Panwar *et al*., 2013a,b). Here, we test the efficacy of stably integrated HD‐RNAi constructs in transgenic wheat plants to achieve resistance against WLR disease by silencing the *PtMAPK1* or *PtCYC1* genes in *Pt*. We show that transgenic expression of HIGS cassettes targeting *Pt* genes provides a viable means to reduce WLR infection in otherwise highly susceptible wheat plants, providing an attractive and precise genetic engineering strategy for combating aggressive rust pathogens.

## Results

### Generation and analysis of transgenic wheat lines for *Pt* resistance

The HD‐RNAi constructs hp‐*PtMAPK1*RNAi and hp‐*PtCYC1*RNAi designed to express the 520‐nt and 501‐nt long 3′‐end coding segments of the fungal *PtMAPK1* and *PtCYC1* genes, respectively, as hairpin loop dsRNA (Experimental procedures) were used for wheat transformation. These constructs were introduced into scutellar tissue of immature embryos of *Pt* susceptible wheat cultivar Fielder by microprojectile bombardment. The HD‐RNAi cassettes were highly specific to the target fungal genes with no discernible homology to off‐target sequences in available wheat genomic resources (Panwar *et al*., 2013a). Fourteen to twenty independent transgenic T_0_ wheat lines harbouring the hp‐*PtMAPK1*RNAi and hp‐*PtCYC1*RNAi construct, respectively, were generated and selfed to obtain the T_1_ seeds. Integration of the *PtMAPK1* or *PtCYC1* gene segment in T_1_ lines was verified by polymerase chain reaction (PCR), and expression of the dsRNA was confirmed by reverse‐transcription PCR (RT‐PCR), using *Pt* gene‐specific primers (Figure S1, Table S1). No visible physiological or growth alterations were observed in the transgenic wheat lines expressing the respective HD‐RNAi construct when compared with the nontransformed control plants of the same genotype, confirming no unintended RNAi effect in the host.

**Figure 1:**
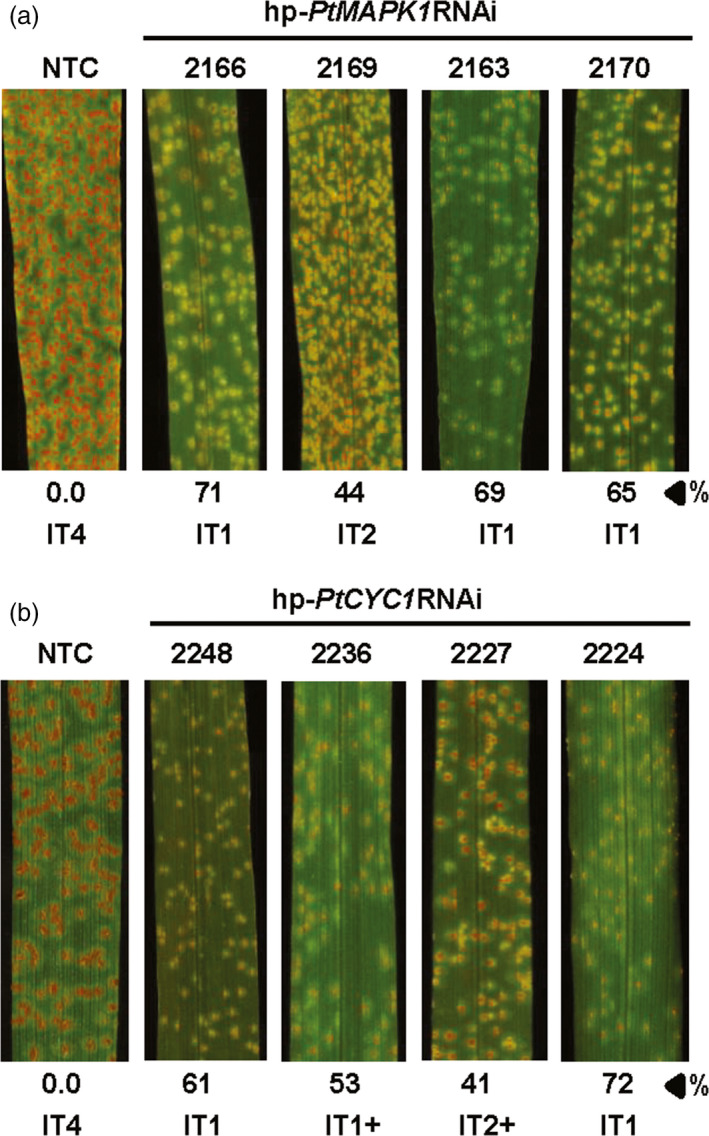
Leaf rust disease resistance in transgenic wheat T_1_ lines. *Pt*‐inoculated leaves of representative transgenic wheat T_1_ lines expressing the hp‐*PtMAPK1* RNAi (a) or hp‐*PtCYC1* RNAi (b) construct show suppressed disease development with low infection types (IT), compared to nontransformed control (NTC) plants. %, represents per cent fungal biomass reduction by HD‐RNAi in a specific transgenic plant selected from the lines shown compared to control. Photographs were taken at 12 dpi.

To examine whether the presence of hp‐*PtMAPK1*RNAi or hp‐*PtCYC1*RNAi constructs in wheat lines affected the susceptibility to infection by *Pt*, 2‐week‐old transgenic T_1_ plants along with nontransformed control plants were challenged with fungal urediniospores and visually observed for WLR development. Analysis of disease phenotype identified six hp‐*PtMAPK1*RNAi and seven hp‐*PtCYC1*RNAi lines that were variably effective in restricting the magnitude of WLR symptoms as compared to controls. Segregation analysis revealed that the fungal transgenes segregated in a Mendelian ratio in these lines, indicating that integration of functional inserts occurred at one (3:1 ratio) or two (15:1 ratio) genetic loci (Table S2). The disease response of *Pt*‐inoculated wheat plants in terms of infection type (IT) was scored at 12 days postinfection (dpi) using the 0–4 scale (Dakouri *et al*., 2013) to reflect uredinia size and abundance. Resistance to *Pt* in T_1_ plants expressing the respective HD‐RNAi constructs was characterized by the appearance of fewer and smaller uredinia with repressed sporulation, corresponding to low infection types varying from 1 to 3. In contrast, *Pt*‐challenged control wheat plants consistently exhibited vigorously sporulating uredinia resembling high ITs, indicative of highly susceptible reactions (Figure 1). Interestingly, the suppression of infection by *Pt* in transgenic wheat plants with low ITs (IT < 2) was also notable for delayed appearance of symptoms as compared to controls. WLR symptoms in control plants became apparent at 5–6 dpi which eventually progressed to large and more distinct uredinia, whereas in transgenic T_1_ plants displaying a low IT, appearance of disease symptoms was delayed by 1–3 days, compared to controls, with markedly reduced uredinia size and density (Figure S2). Taken together, a quantitative reduction of WLR disease symptoms was observed in transgenic wheat lines carrying a HIGS construct targeting the fungal *PtCYC1* or *PtMAPK1* gene.

### Transgenic wheat lines expressing the HD‐RNAi constructs impede *Pt* development

**Figure 2:**
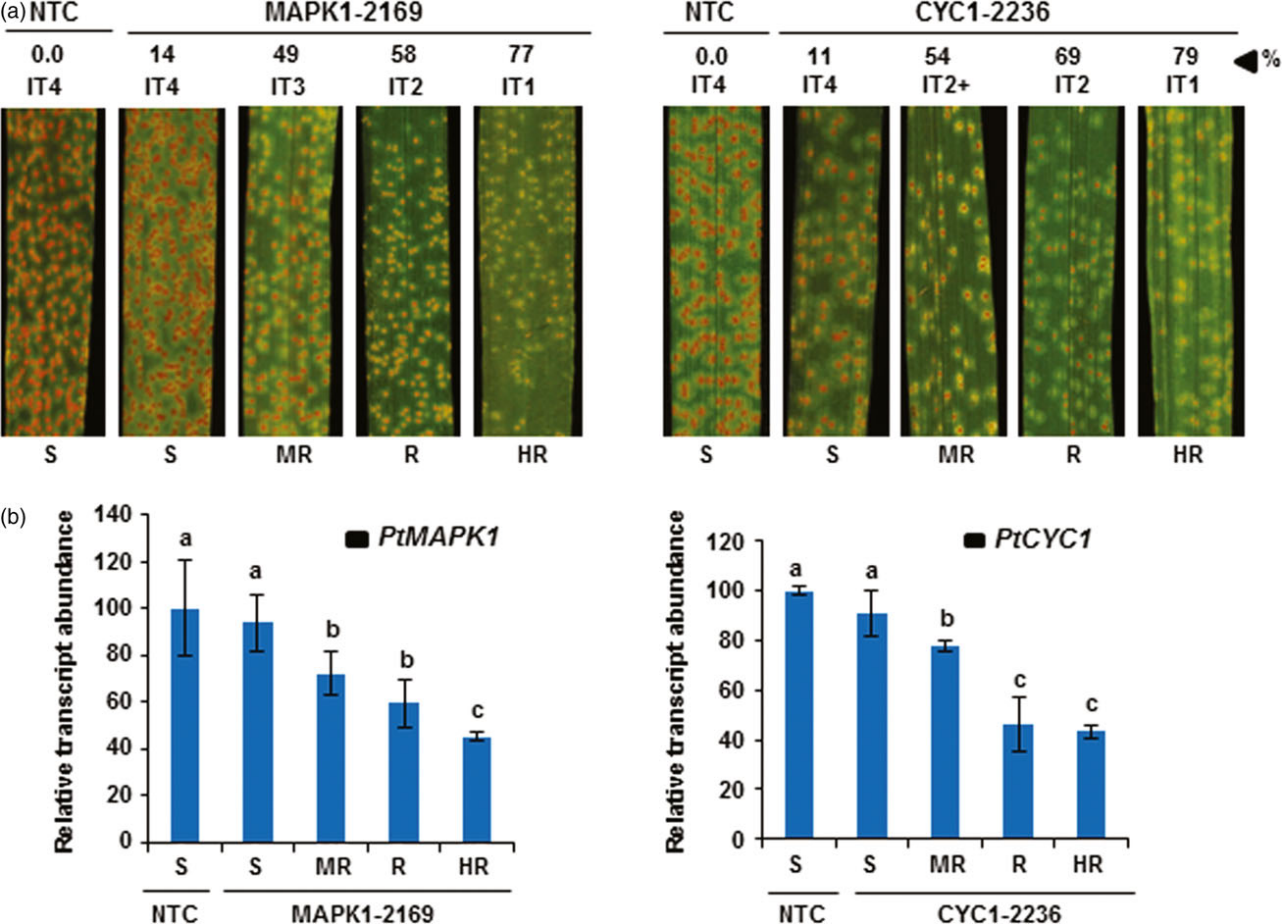
HD‐RNAi of *PtMAPK1* and *PtCYC1* genes in *Pt* infecting transgenic wheat plants. (a) Disease symptoms on transgenic wheat T_1_ plants expressing the hp‐*PtMAPK1* RNAi (line 2169) or hp‐*PtCYC1* RNAi (line 2236) construct and nontransformed control (NTC) plants following challenge with *Pt*. Representative leaves are shown from transgenic plants of selected lines classified into highly resistant (HR), resistant (R), moderately resistant (MR) and susceptible (S) categories based on per cent fungal biomass reduction (%) in infected tissue compared to controls, along with the scored infection type (IT). Photographs were taken at 12 dpi, the same time point when fungal biomass ratios were analysed by qPCR. (b) Quantitation of fungal *PtMAPKI* or *PtCYC1* transcript abundance in *Pt*‐inoculated leaves of transgenic T_1_ plants of lines MAPK1‐2169 and CYC1‐2236 representing different resistance types (as presented in a) by RT‐qPCR at 5 dpi. The expression levels of *PtMAPK1* or *PtCYC1* were normalized to the levels of the *Pt* succinate dehydrogenase gene and are reported as a percentage, relative to that of the NTC plants (set at 100%). A correlation was observed between HIGS‐derived relative reduction in *PtMAPK1* and *PtCYC1* transcript levels and per cent fungal biomass reduction (% in a). Bars represent mean values ± SDs of two independent experiments. Values with different letters indicate statistically significant differences (Student's *t*‐test; *P* < 0.05).

We next explored whether suppression of *Pt* development in transgenic wheat T_1_ lines was associated with impairment of fungal growth inside leaf tissue by measuring the fungal biomass accumulation in leaves challenged with *Pt*. Determination of fungal biomass showed that the ratio of fungal‐to‐plant DNA was clearly, but varyingly, reduced in transgenic lines, compared to nontransformed controls (Table 1). The hp‐*PtMAPK1*RNAi expressing plants of lines MAPK1‐2163, MAPK1‐2169 and MAPK1‐2166 were the most effective in restricting fungal development, showing biomass reductions by up to 77% in comparison with controls (Figures 1a and 2a). Similarly, fungal biomass was reduced by as much as 79% in hp‐*PtCYC1*RNAi expressing plants of lines CYC1‐2236, CYC1‐2248 and CYC1‐2224 as compared to controls (Figures 1b and 2a). This marked reduction in fungal load in T_1_ lines suggested that the presence of the HD‐RNAi constructs in transgenic wheat plants interfered with *Pt* virulence and thereby attenuating the capability of the fungus to colonize the host tissue.

**Table 1:** Response of transgenic wheat T_1_ lines to *Pt* infection

HD‐RNAi construct and line number	Disease intensity based on reduction in fungal biomass				
	*n*	S	MR	R	HR
hp‐*PtMAPK1*RNAi					
MAPK1‐2159	15	8	5	2	–
MAPK1‐2162	12	7	4	1	–
MAPK1‐2163	12	8	2	1	1
MAPK1‐2166	23	9	9	5	–
MAPK1‐2169	18	9	4	4	1
MAPK1‐2170	16	7	6	3	–
Fielder‐NTC	15	15	–	–	–
hp‐*PtCYC1*RNAi					
CYC1‐2216	14	6	5	3	–
CYC1‐2224	15	10	2	2	1
CYC1‐2226	6	4	2	–	–
CYC1‐2227	11	3	5	3	–
CYC1‐2228	14	9	2	3	–
CYC1‐2236	15	9	3	2	1
CYC1‐2248	13	6	4	3	–
Fielder‐NTC	15	15	–	–	–

*n*, number of plants tested (PCR positive for transgenic lines); NTC, nontransgenic control. HR, highly resistant, fungal biomass reduced by more than 75%; R, resistant, fungal biomass reduction by 50%–75%; MR, moderately resistant, reduction in fungal biomass by 25%–50%; S, susceptible, reduction in fungal biomass by <25% as compared to control plants.

Ratio of fungal‐to‐wheat DNA analysed by qPCR using total genomic DNA extracted from *Pt*‐inoculated leaves at 12 dpi.

Generally, resistance to rust pathogens is not an apparent case of immunity versus susceptibility but, more often, is perceived as a quantitative difference in fungal growth between more or less resistant and susceptible plants which makes visual disease scoring systems sporadically prone to variability and error. To circumvent this possibility, we grouped transgenic T_1_ HD‐RNAi plants into four different resistance types based on the quantification of fungal biomass in *Pt*‐infected tissue by qPCR. The rationale behind such classification was to ensure accurate and unbiased disease resistance pattern detection in transgenic lines. Transgenic plants were considered as highly resistant (HR) to *Pt* if the HIGS‐derived reduction in fungal biomass was more than 75% relative to control plants, reduction in fungal biomass by 50%–75% was designated as a resistant (R) phenotype, 25%–50% reduction in fungal biomass was classified as moderately resistant (MR), whereas plants in which fungal biomass accumulation was suppressed by <25% as compared to controls were categorized as susceptible (S; Table 1). Overall, we observed a close relationship between WLR disease severity and the amount of fungal biomass detected in *Pt*‐challenged wheat plants. For example, wheat plants with visibly high disease symptoms (high ITs) also showed higher fungal biomass in infected leaves than those suppressing fungal development (low ITs) that had less fungal biomass (Figure 2a).

### HD‐RNAi results in reduction of targeted gene transcript levels in *Pt* infecting transgenic wheat lines

**Figure 3:**
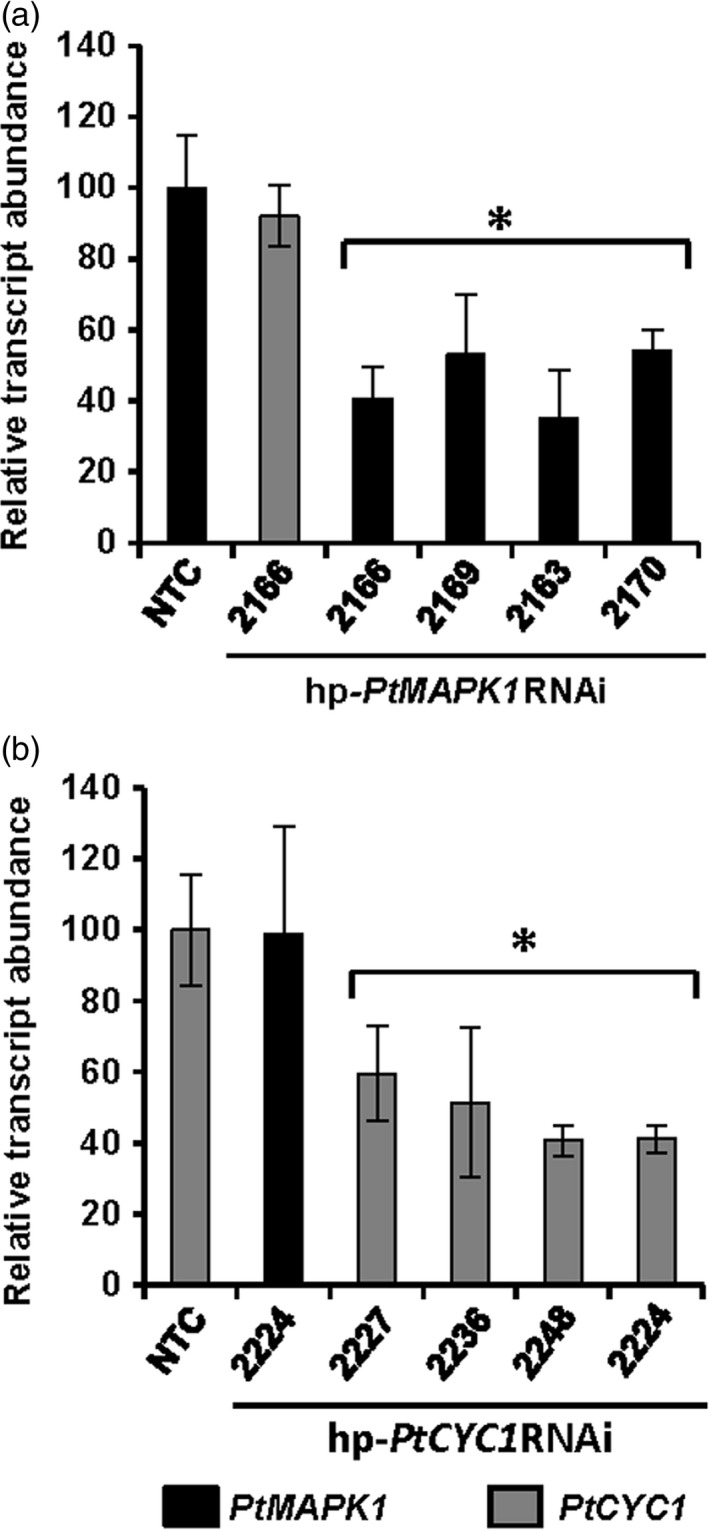
HIGS‐derived silencing of targeted *Pt* genes in transgenic wheat T_1_ lines. RT‐qPCR analyses show significant reduction in transcript abundance of fungal *PtMAPK1* or *PtCYC1* genes in selective transgenic wheat T_1_ lines expressing the respective hp‐*PtMAPK1* RNAi (a) and hp‐*PtCYC1* RNAi (b) construct as compared to nontransformed control (NTC) plants. cDNA was generated from total RNA isolated from *Pt*‐challenged leaves at 5 dpi. The expression levels were normalized to the level of the endogenous *Pt* reference succinate dehydrogenase gene with control set at 100%. No HD‐RNAi effect is seen on the other, sequence‐unrelated target fungal gene. Bars represent mean values ± SDs of three independent sample collections, each using leaves pooled from an average 20–25 infection sites of 4–7 plants. *Statistically significant difference with controls, at *P* < 0.05 (Student's *t*‐test).

In order to substantiate the hypothesis that protection to WLR infection in hp‐*PtMAPK1*RNAi or hp‐*PtCYC1*RNAi lines was caused by *in planta*‐derived silencing of the target *PtMAPK1* or *PtCYC1* genes, we quantified the transcript levels of the respective genes in *Pt* infecting transgenic T_1_ and nontransformed control wheat plants at 5 dpi. Transcript levels of the *PtMAPK1* and *PtCYC1* genes were reduced by approximately 41%–65% in *Pt* invading transgenic wheat lines expressing the complementary HD‐RNAi constructs compared to *Pt*‐infected controls (Figure 3). This indicated that integration of HD‐RNAi expression cassettes in transgenic wheat was sufficient to interfere with transcription of the targeted fungal genes during infection. The endogenous silencing of the target fungal genes in transgenic wheat plants was specific to the presence of the corresponding HD‐RNAi construct because no effect was observed on the expression of the *PtCYC1* gene in the hp‐*PtMAPK1*RNAi lines and *vice versa* (Figure 3). These findings support the conclusion that *PtMAPK1* and *PtCYC1* contribute to *Pt* virulence and knockdown of their expression by HIGS compromised the fungus ability to proliferate in transgenic wheat.

Next, we asked whether there was an association between HIGS‐derived reduction of *PtCYC1* or *PtMAPK1* transcript levels in *Pt* and the growth penalty imposed on the fungus in transgenic wheat HD‐RNAi lines. Two lines, MAPK1‐2169 and CYC1‐2236, one from each hp‐*PtMAPK1*RNAi and hp‐*PtCYC1*RNAi transgenic events, were selected, and the relative abundance of fungal *PtCYC1* or *PtMAPK1* transcripts was measured in transgenic plants representing the range of four different resistance types and compared to levels present in *Pt* infecting control plants. The analysis showed a close relationship between the endogenous *PtMAPK1* and *PtCYC1* transcript levels in the fungus invading transgenic wheat plants expressing the corresponding HD‐RNAi construct and the amount of fungal biomass accumulating within the infected host tissues (Figure 2b). The highest reduction in fungal *PtMAPK1* or *PtCYC1* transcript abundance by HD‐RNAi was observed in transgenic plants that also displayed the lowest fungal biomass accumulation (rated as HR) followed by resistant and moderately resistant plants. These results indicate that the effectiveness of HD‐RNAi to thwart WLR infection in transgenic wheat plants was associated with the silencing efficiency of the targeted genes in the colonizing fungus.

### Resistance to *Pt* in transgenic wheat persists in the advanced generation

**Figure 4:**
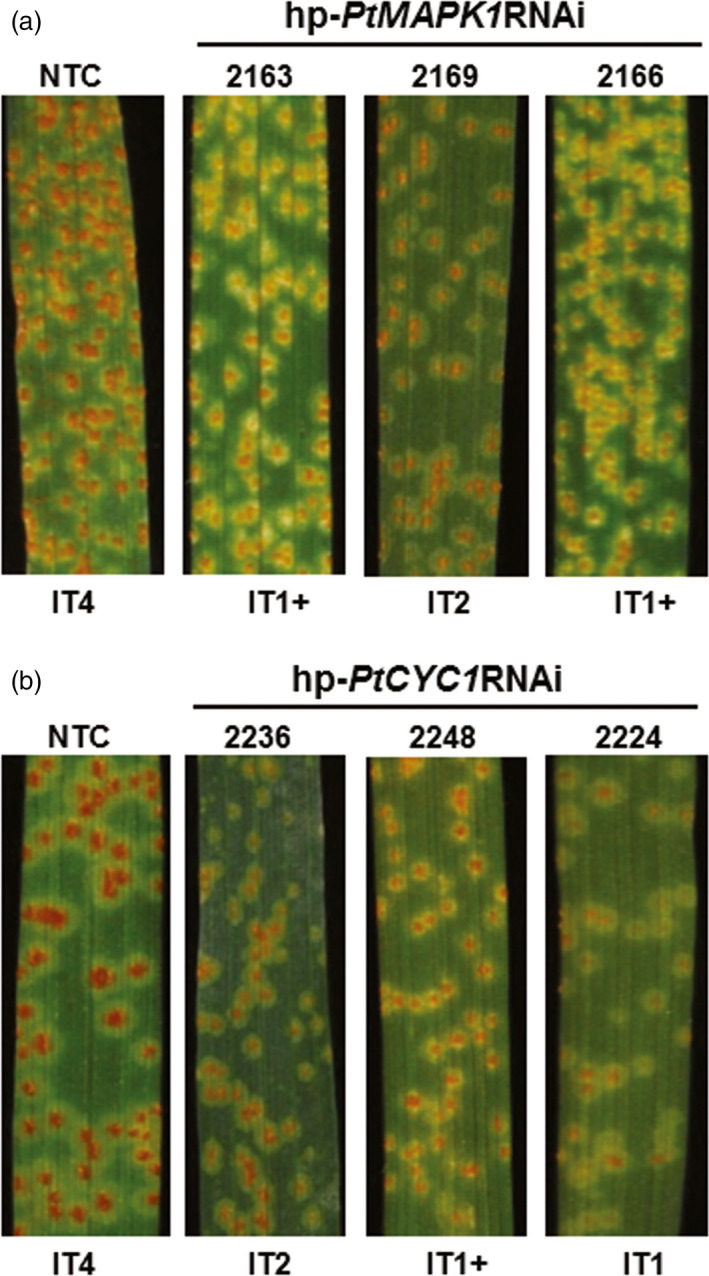
Leaf rust disease resistance in transgenic wheat T_2_ lines. *Pt*‐inoculated leaves of representative transgenic wheat T_2_ lines expressing the hp‐*PtMAPK1* RNAi (a) or hp‐*PtCYC1* RNAi (b) construct show suppressed disease symptoms with low infection types (IT), compared to nontransformed control (NTC) plants. Photographs were taken at 12 dpi.

To investigate the impact of HD‐RNAi on suppression of *Pt* infection in advanced generations, three transgenic wheat lines for each of the hp‐*PtMAPK1*RNAi (MAPK1‐2163, MAPK1‐2169 and MAPK1‐2166) and hp‐*PtCYC1*RNAi (CYC1‐2236, CYC1‐2248 and CYC1‐2224) construct that showed enhanced disease protection were selected and selfed to obtain T_2_ progeny. PCR and RT‐PCR screening revealed the stable inheritance and expression of respective HD‐RNAi constructs in transgenic wheat T_2_ plants (Figure S3). Resistance to *Pt* was assessed by inoculating 2‐week‐old transgenic and control wheat plants with fungal urediniospores. Disease phenotype analyses showed that WLR development was radically altered in transgenic wheat T_2_ lines, as indicated by fewer pustules of much smaller size often surrounded by chlorotic or necrotic halos. In contrast, leaves of *Pt*‐infected control plants were completely overgrown by large sporulating uredinia (Figure 4). The disease lesions evaluated at 12 dpi were mainly scored as low IT (IT 1–3) in the T_2_ plants, reflecting restricted *Pt* proliferation, which was substantially less severe than the highly susceptible phenotype (IT4) that was consistently observed in control plants. Also, a delay in both the onset and progression of WLR symptoms was seen in transgenic T_2_ plants with low ITs (IT < 2) as compared to control plants (Figure S4). These results suggested that HIGS of *PtMAPK1* or *PtCYC1* genes was effective in limiting the growth of pathogenic *Pt* in transgenic wheat T_2_ lines.

**Figure 5:**
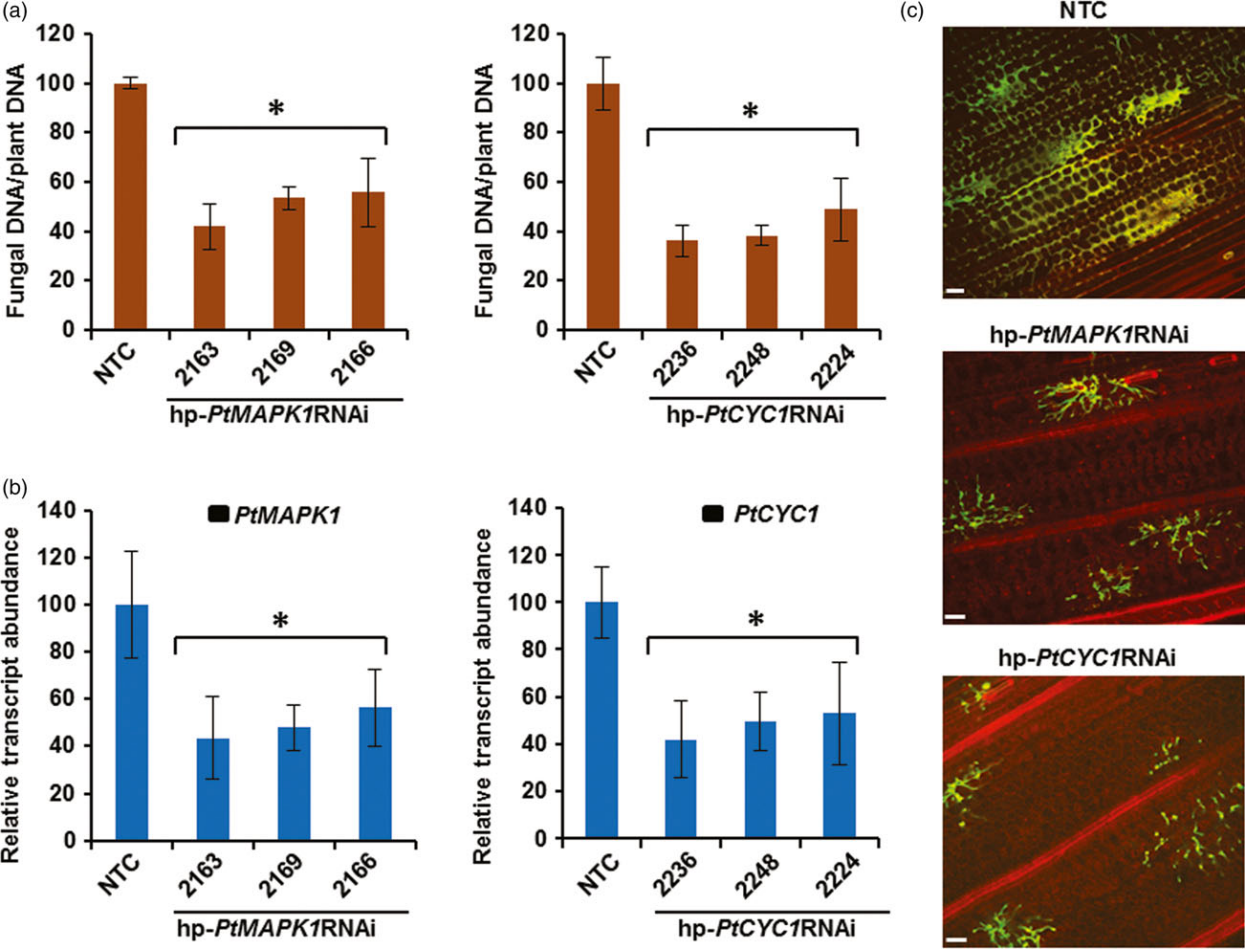
Quantitative protection from *Pt* infection in transgenic wheat T_2_ lines carrying HD‐RNAi constructs. (a) Fungal biomass measurements determined by qPCR in *Pt*‐challenged leaves of hp‐*PtMAPK1* RNAi or hp‐*PtCYC1* RNAi expressing transgenic wheat lines and nontransformed control (NTC) plants at 12 dpi. (b) Relative transcript abundance of target *PtMAPK1* or *PtCYC1* genes analysed by RT‐qPCR in *Pt*‐challenged transgenic wheat T_2_ lines expressing the corresponding hp‐*PtMAPK1* RNAi or hp‐*PtCYC1* RNAi constructs and NTC plants. cDNA was generated from total RNA isolated at 5 dpi. The expression levels were normalized to the level of the endogenous *Pt* reference succinate dehydrogenase gene, with control set at 100%. Bars represent mean values ± SDs of three independent sample collections, each using leaves pooled from an average 20–25 infection sites of 4–7 plants. *Statistically significant difference with controls, at *P* < 0.05 (Student's *t*‐test). (c) Confocal microscopy of *Pt* infection in wheat leaves at 5 dpi, showing restricted mycelial development in transgenic samples expressing the respective HD‐RNAi construct as indicated, compared to the control plant. Bars: 70 μm.

To quantify the inhibitory effect of HD‐RNAi on fungal development, the amount of fungal DNA in *Pt*‐inoculated leaf samples of transgenic T_2_ and control wheat plants was measured by qPCR. As expected, transgenic T_2_ lines accumulated substantially reduced fungal biomass averaging between 44% and 64%, compared to control plants (Figure 5a). RT‐qPCR analysis confirmed that *in planta* inhibition of fungal growth in transgenic T_2_ lines was due to silencing of targeted genes in *Pt*. Silencing induced by HD‐RNAi significantly reduced *PtMAPK1* and *PtCYC1* relative transcript levels by approximately 44%–58% in *Pt* colonizing the corresponding transgenic wheat T_2_ line, compared to the normal expression levels detected in fungi infecting control plants (Figure 5b). Microscopic examination of *Pt* development in infected tissue revealed normal mycelial morphology with extensive colonization of host cells in control plants, whereas hyphal growth was significantly compromised in transgenic lines (Figure 5c). This suggests that depletion of target fungal gene transcripts by HD‐RNAi imposed a growth arrest on invading *Pt* in its transgenic host. Taken together, these results showed that HD‐RNAi constructs targeting the *PtMAPK1* or *PtCYC1* gene in *Pt* were able to confer genetically stable resistance to WLR infection in transgenic wheat.

### Production of siRNAs in transgenic wheat

**Figure 6:**
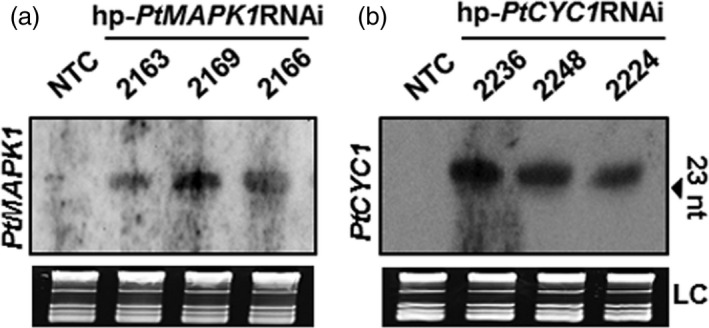
siRNA detection in transgenic wheat lines. RNA blots showing the presence of *PtMAPK1* (a) and *PtCYC1* (b) sequence‐specific siRNA molecules in transgenic wheat T_2_ lines expressing the respective hp‐*PtMAPK1* RNAi or hp‐*PtCYC1* RNAi construct. Total RNA was extracted from wheat leaf tissue prior to fungal inoculations. RNA blots were hybridized with target fungal gene‐specific DIG‐labelled probes. Ethidium bromide‐stained rRNA serve as loading controls (LC). The arrow indicates oligonucleotide marker size. NTC, nontransformed control.

We examined whether the silencing of the targeted *PtMAPK1* and *PtCYC1* genes in *Pt* by HD‐RNAi was associated with the presence of homologous siRNAs in transgenic wheat plants. Northern blot analysis of total RNA extracted from transgenic T_2_ wheat lines expressing the respective hp‐*PtMAPK1*RNAi or hp‐*PtCYC1*RNAi construct prior to *Pt* inoculation revealed the presence of corresponding siRNA molecules (Figure 6). No detectable hybridization signals were observed in nontransformed control plants. The findings showed that hp‐*PtMAPK1*RNAi or hp‐*PtCYC1*RNAi sequences expressed in transgenic wheat plants were processed by the host silencing machinery into siRNA molecules, and these probably are translocated to fungal cells during infection to act upon the target endogenous mRNAs in a nucleotide‐specific manner thereby inhibiting their function in the fungus.

## Discussion

In this study, we have shown that developing transgenic wheat plants stably expressing HD‐RNAi constructs designed to target *Pt* genes that are crucial for fungal pathogenicity is an effective strategy to obtain enhanced resistance against the destructive WLR disease. The constitutive expression of HD‐RNAi constructs in transgenic wheat lines induced efficient silencing of the targeted *PtMAPK1* or *PtCYC1* genes in the interacting *Pt* resulting in subsequent impaired fungal development and a reduction in disease severity and progression in otherwise highly susceptible wheat plants. The engineered resistance trait was heritable and stable in the T_2_ generation. Suppression of WLR development was correlated with the presence of siRNA molecules specific to the fungal *PtMAPK1* or *PtCYC1* genes in the transgenic wheat lines expressing the complementary HD‐RNAi construct. The *in vivo* reduction in *PtMAPK1* and *PtCYC1* transcript levels in *Pt* feeding on transgenic wheat leaves expressing the homologous siRNA molecules indicated the manifestation of PTGS by HIGS.

The MAP‐kinase and cyclophilin‐encoding genes were selected for the transgenic HD‐RNAi approach because they are implicated in playing fundamental roles in the regulation of various physiological and pathogenesis processes in eukaryotes (Wang and Heitman, 2005). In addition, we demonstrated previously by transient HIGS assays that *PtMAPK1* and *PtCYC1* genes are important for pathogenicity in *Pt* (Panwar *et al*., 2013a,b). Cyclophilins have been reported to act as virulence determinants in diverse plant pathogenic fungi such as *Magnaporthe grisea* (Viaud *et al*., 2002), *Botrytis cinerea* (Viaud *et al*., 2003) and *Cryphonectria parasitica* (Chen *et al*., 2011). MAP‐kinases from phytopathogenic fungi have also been extensively studied and shown to regulate cell wall biogenesis, morphogenesis, pathogenic development and disease (Martínez‐Soto and Ruiz‐Herrera, 2015; Zhao *et al*., 2007). Earlier, it was reported that the *PtMAPK1* gene could complement an *Ustilago maydis MAPK* (*kpp2 kpp6*) double deletion mutant to restore mating and full virulence (Hu *et al*., 2007a). Similarly, *Fus3/Kss1‐type MAPK* from *Pst*, when expressed in ascomycete *Fusarium graminearum* and *M. oryzae*, complemented the corresponding *kss1* and *pmk1* mutants to partially restore vegetative growth and ability to infect plants (Guo *et al*., 2011). Consistent with the well‐defined functions of MAP‐kinases and cyclophilins in ensuring pathogen ability to cause disease, we have shown that silencing their homologs in *Pt* by transgenic HD‐RNAi significantly protected host wheat plants from the disease.

An obligatory requirement for HIGS is the mobility of silencing signals from host plant species into the invading pathogen. The reduction in endogenous transcript abundance of *PtMAPK1* and *PtCYC1* genes in *Pt* by the respective HD‐RNAi construct was associated with the presence of complementary siRNAs in the transgenic wheat plants. This indicated that these could be the silencing molecules that initiated *in vivo* RNAi of the corresponding *PtMAPK1* and *PtCYC1* genes in the colonizing fungus. Recently, siRNAs have been reported to move across interacting organisms, encompassing cross‐species silencing and communication between host and pathogens or parasites (Knip *et al*., 2014; Weiberg *et al*., 2015). A correlation between HIGS in phytopathogenic fungi and the presence of pathogen gene‐specific siRNAs in host plants was also observed in other diverse interactions, suggesting that there is a successful translocation of siRNAs from host plants to parasitic fungi (Andrade *et al*., 2015; Ghag *et al*., 2014; Govindarajulu *et al*., 2015; Nowara *et al*., 2010; Tinoco *et al*., 2010). In the wheat–rust pathosystem, the HD‐RNAi signals may traverse from host to fungus via the haustorial interphase (Panwar *et al*., 2013a) utilizing the mechanism of vesicle‐mediated (exosomes) transport (Knip *et al*., 2014). Despite the progress being made, several aspects of the trans‐kingdom RNAi are still poorly understood and more extensive research is required to validate the nature of silencing signals (whether siRNA or precursor dsRNA; e.g., Koch *et al*., 2016) and their route of delivery during plant–pathogen interactions.

Inhibition of WLR infection in transgenic wheat lines demonstrated that the degree of silencing achieved by HIGS of the target *PtMAPK1* and *PtCYC1* genes was sufficient to attenuate fungal pathogenicity. Although the severity of disease was significantly compromised in transgenic wheat leaves, we did not observe complete protection against *Pt* as few pustules continued to develop from initial infection sites. This can be attributed to partial silencing of the *PtMAPK1* or *PtCYC1* gene as seen in incomplete knockdown of the target fungal transcripts (Figure 3 and 5b); it is possible that the presence of residual transcripts was sufficient to confer some level of virulence. While RNAi can inhibit transcript expression, it normally does not completely eliminate gene function (Eamens *et al*., 2008). The fact that we observed variability from R to S in the resistance response within transgenic lines in the T1 generation (Fig. 2 and Table 1) is most likely due to segregation of the transgene locus or loci in this generation. Gene expression levels would be expected to vary between plants where the transgene locus is homozygous vs heterozygous which could explain why some plants are scored as R and some as MR, and segregants lacking the transgene will be scored as S. Other factors possibly affecting HIGS efficiency could be the abundance and turnover rate of target mRNAs. Nevertheless, we observed that the efficacy of HD‐RNAi constructs in reducing fungal growth in transgenic wheat lines correlated well with the reduced levels of the corresponding *PtMAPK1* and *PtCYC1* gene transcripts in the invading fungus. A similar cause–effect relationship between HIGS‐derived depletion of pathogen mRNA levels and impairment of host infection competency was also reported in different fungal–plant pathosystems (Andrade *et al*., 2015; Koch *et al*., 2013). These results indicate that, when exploring HIGS to generate effective plant disease resistance, the extent of target gene silencing in the pathogen is likely to be important. However, the choice of effective candidate genes is arguably the most crucial parameter for the success of this technology (Panwar *et al*., 2016).

The WLR disease protection in the transgenic wheat lines was marked by reduced uredinia density and size and by a general delay in symptom appearance in infected host tissue. This supports the notion that the HD‐RNAi effect against *Pt* was quantitative in nature. From a breeding point of view, partial resistance is often regarded as more durable, as it can potentially limit the trade‐offs between virulence and reproductive fitness in pathogen population, thereby preventing the emergence of a virulent race (Burdon *et al*., 2014). Indeed, in our transgenic plants, reduced sporulation would decrease fungal population size in the field. In contrast, rust disease control measures that rely on exploiting major resistance genes that recognize pathogen effectors or their actions have often proved to be ephemeral owing to rapid adaptations by these fungi to escape recognition in resistant plants (Dangl *et al*., 2013; Stokstad, 2007). In this regard, targeting pathogen genes that are indispensable for morphogenesis and/or pathogenesis by HD‐RNAi can presumably provide long‐lasting protection because modifying or deleting such essential genes would likely result in major consequences to the pathogen. To increase the degree of functional constraints facing the pathogen upon infection, an effective HIGS design can be silencing multiple genes that are required for pathogenicity rather than relying on single gene targets. In a recent study, simultaneous silencing of three cytochrome P450 lanosterol C‐14α‐demethylase (*CYP51*) genes in *F. graminearum* by HD‐RNAi was shown to completely restrict fungal infection in transgenic *Arabidopsis* and barley (Koch *et al*., 2013), although multiple targets may not always be more effective (Chen *et al*., 2016). Therefore, one approach would be crossing the hp‐*PtMAPK1*RNAi and hp‐*PtCYC1*RNAi lines to investigate whether or not a cumulative effect would result in more *Pt*‐resistant lines.

Earlier, transient HD‐RNAi assays were utilized for analysing gene function in the wheat rust pathogens (Panwar *et al*., 2013a,b; Yin *et al*., 2015; Zhang *et al*., 2012). A major limitation of transient RNAi techniques, despite being useful in fast forward functional genomic platforms, is that they elicit uneven and short‐lived silencing responses (Bhaskar *et al*., 2009; Holzberg *et al*., 2002) which are not suitable for sustainable applications of HIGS in agriculture. Stable HD‐RNAi on the other hand should offer many advantages such as the establishment of more uniform gene silencing and the transmission of disease resistance traits to next generations. Production of transgenic crop plants will therefore provide a genetic resource to harvest the full potential of HIGS for engineering long‐lasting resistance against rust diseases. However, a drawback of transforming a monocotyledonous species, such as wheat, is that it is an arduously lengthy and random process, making it practically unsuitable for screening large number of genes to identify efficient HIGS targets. Therefore, a rational approach would be to first validate the function of potential candidate genes identified in the pathogen by transient HD‐RNAi approaches and those showing the ability to deter parasitism in the host can then be tested for the development of transgenic disease‐resistant varieties.

The ability to express HIGS constructs in transgenic plants for control of plant pathogens offers an attractive strategy for managing pests of significant importance (Knip *et al*., 2014). However, the effectiveness of HD‐RNAi to impart genetically stable resistance against rust pathogens has not been explored. An important issue to investigate in the future is whether in subsequent wheat generations RNA‐mediated DNA methylation of the silencing constructs may occur, thereby reducing transgene expression levels. Our study provides proof of concept that HIGS against rust fungi can be made operational in stable transgenic plants and results are in line with, and extend beyond, technical advancements made in recent years over the potential application of this technology for fighting rust infections in cereal crop plants (Panwar *et al*., 2013a,b; Yin *et al*., 2014). The lack of homology between the chosen *Pt* sequences and published wheat genomic sequences ensured that the likelihood of any off‐target silencing effect against a host gene was low; indeed, we did not observe any sort of deviation from a normal growth phenotype in the transgenic wheat plants. By the same analogy though not directly tested here, the transgenic wheat hp‐*PtMAPK1*RNAi and hp‐*PtCYC1*RNAi lines will likely also prove effective against many other leaf (*Pt*), stem (*Pgt*) and stripe (*Pst*) rust isolates as the sequences used in the HD‐RNAi constructs have high homology to the corresponding gene sequences in other sequenced isolates and species. Earlier, we showed through extensive molecular studies using the same constructs in the transient system that the suppression of disease symptoms was highly correlated with the silencing of the target genes and reduction in fungal biomass for various different isolates from all three rust species (Panwar *et al*., 2013a). Due to complexities associated with conducting reverse genetics studies in rust pathogens, to date, the role of only a few fungal genes involved in the infection processes has been identified. Given that HIGS has emerged as a promising method to analyse gene function in otherwise genetically recalcitrant rust fungi, more studies are needed to address the roles of the large repertoires of predicted pathogenicity‐related genes available from genome sequencing projects. The use of such virulence‐determining or essential genes that might be functionally difficult to overcome by pathogens promises a potential avenue towards sustainable rust disease protection in wheat cultivars.

## Experimental procedures

### Plant materials, *Pt* inoculations and disease assessment

Leaf inoculations were performed using *Pt* isolate CCDS. Urediniospores were increased on susceptible wheat cultivar Fielder plants. The inoculum was prepared by suspending urediniospores in light mineral oil (Baytol) at a rate of 12 mg/mL. Inoculum was applied to the leaves of 2‐week‐old wheat plants using an air brush. The inoculated plants were transferred to a dark dew chamber preconditioned at 22 °C and 100% relative humidity. After 18 h of incubation, plants were transferred to the greenhouse and grown under 16:8 h day: night regime at 22 °C. Disease development in terms of infection types (IT) was assessed when rust symptoms were fully expressed at 12 dpi using the seedling 0–4 scale (Dakouri *et al*., 2013). In short, infection types 0: no flecks or uredinia; IT 1: small uredinia with necrosis; IT 2: small‐to‐medium uredinia with necrosis: IT 3: moderate‐to‐large size uredinia with/without chlorosis; IT 4: very large uredinia. The variation within each class is indicated by the use of – (less than average) and + (more than average).

### Construction of HD‐RNAi vectors

The *Pt* genes encoding a MAP‐kinase (*PtMAPK1*) and a cyclophilin (*PtCYC1*) that we earlier characterized to have an essential role in pathogenicity were used in this study (Panwar *et al*., 2013a). Binary vectors for wheat transformation were constructed as described earlier (Panwar *et al*., 2013a). Briefly, a 520‐bp fragment representing the 3′‐end coding region of the *PtMAPK1* gene (Hu *et al*., 2007a) GenBank accession #68303937) was PCR‐amplified from cDNA of *Pt* Race 1 isolate BBBD using primers *PtMAPK1*‐F and *PtMAPK1*‐R (Table S1). The amplified segment was cloned into pENTER™ vector using the D‐TOPO® Cloning kit (Invitrogen, Carlsbad, CA) and the recombinant entry module recombined in the cereal‐specific binary vector pIPK007 (Himmelbach *et al*., 2007) to generate hp‐*PtMAPK1*RNAi. The resulting RNAi vector allowed expression of hpRNA molecules under the control of the *Zea mays* ubiquitin promoter and terminator sequences. To generate hp‐*PtCYC1*RNAi, a 501‐bp 3′‐end coding region of *PtCYC1* (Hu *et al*., 2007b) GenBank accession #BU672663) was amplified from the EST sequence (*Pt*Contig6674) using primers *PtCYC1*‐F and *PtCYC1*‐R (Table S1). The amplified fragment was sequentially cloned into plasmid pUBleX1‐RNAi to generate an intron‐spliced hpRNA segment that was subsequently cloned into the binary vector pMCG161 (http://www.chromdb.org/rnai/vector_info.html) under the control of the *Cauliflower mosaic virus* 35S promoter and octopine synthase terminator. All final constructs were verified by sequencing.

### Genomic DNA extraction

Fungal genomic DNA was extracted from germinating urediniospores as described earlier (Panwar *et al*., 2013a). Urediniospores were germinated by dusting them over sterile water in dishes for over 8 h using a volatile nonanol solution [1.56 μL nonanol (Sigma‐Aldrich, ON, Canada), 1 mL acetone, 19 mL of ddH_2_0] spotted on filter paper suspended in the lids to stimulate germination. Plant genomic DNA was extracted from leaf tissue using the DNeasy plant Mini Kit (Qiagen, Mississauga, ON, Canada) as described by the manufacturer.

### Total RNA extraction and cDNA synthesis

Total RNA was extracted using TRIzol reagent (Invitrogen) as described by the manufacturer. The quality and concentration of total RNA were assessed via denaturing 1.2% agarose gel and 260/280ABS measurements on a NanoDrop spectrophotometer (ThermoFisher Scientific, Mississauga, ON, Canada). Total RNA was treated with DNase I using the TURBO DNA‐free™ kit (ThermoFisher Scientific, Mississauga, ON, Canada) following manufacturer instructions to remove DNA contamination prior to cDNA synthesis. The first stand of cDNA was synthesized using 1.0 μg of RNA sample with a poly(A)_18_ oligonucleotide primer using the Reverse Transcriptase III (ThermoFisher Scientific, Mississauga, ON, Canada) according to the manufacturer's protocol.

### Wheat transformation

Plasmid suspensions at a concentration of 1 μg/μL in 10 mm Tris (pH 8.0) were used for wheat transformation by particle bombardment as described earlier (Jordan, 2000) with minor modifications. Immature embryos of wheat cv. Fielder were cultured on Murashige and Skoog medium supplemented with 0.5 mg/L thiamine, 100 mg/L glutamine, 1 mm niacinamide, 2 mg/L 2,4‐D and solidified with 2.5 g/L phytagel (Sigma‐Aldrich). A 2‐day preculture was used prior to bombardment using 0.6‐μm gold, 650 psi rupture discs and a 3 cm target distance. After callus growth on RG5 medium for 14 days, calli were transferred to 1/2 strength MS with 25 mg/L of hygromycin B (Sigma‐Aldrich) for 21 days under a 16:8 h light: dark regime prior to subculture every 14 days on the same selectable medium, followed by planting in soil in growth cabinets to grow to maturity. Integration and expression of HD‐RNAi transgene in transgenic wheat plants were confirmed by PCR and RT‐PCR analysis using candidate *Pt* gene‐specific primers (Table S1). PCRs were subjected to 30 cycles of denaturation (94 °C, 1 min), annealing (58 °C, 30 s) and extension (72 °C, 30 s). The goodness of fit of the observed segregation ratio for the transgene was tested against the Mendelian segregation ratio of 3:1 and 15:1 using the chi‐square (χ^2^) test.

### Quantitative real‐time PCR analysis

Quantitative real‐time PCR analysis was carried out using a CFX96™ Real‐Time PCR machine (Bio‐Rad, Mississauga, ON, Canada). Transcript levels of the target *Pt* genes, *PtMAPK1* and *PtCYC1*, were measured using cDNA prepared from total RNA isolated from wheat leaf tissue inoculated with *Pt* by RT‐qPCR as described previously (Panwar *et al*., 2013a). Specific primers for each gene were designed using the Primer 3.0 program and are shown in Table S1. Quantification results were analysed using the comparative 2−ΔΔCT method. To assess fungal transcript levels, the *Pt* succinate dehydrogenase (SDH) gene was used as normalizing reference gene. To quantify fungal biomass, the ratio of single copy *PtRTP1* and the wheat *TaEF1* genes was assessed in genomic DNA isolated from infected leaves using gene‐specific primers (Panwar *et al*., 2013a). The relative amounts of PCR product of *PtRTP1* and *EF1* in *Pt*‐infected samples were calculated using generated gene‐specific standard curves to quantify the *Pt* and wheat gDNA, respectively.

### Confocal microscopy

Microscopic analysis of mycelium growth in *Pt*‐inoculated wheat leaves was carried out using a Leica SP2‐AOBS laser scanning confocal microscope (Leica, Mannheim, Germany) at 5 dpi. Fungal structures were stained with Uvitex‐2B (Sigma‐Aldrich), and Acridine Orange (Sigma‐Aldrich) was used to stain plant cell walls following procedures as described previously (Panwar *et al*., 2013b). The fluorescence of Uvitex‐2B and Acridine Orange was detected by excitation at 405 and 514 nm, and scanning was performed with filter setting at 411–485 and 550–560 nm, respectively.

### siRNA detection

Small RNA detection was performed using total RNA extracted from wheat leaves prior to *Pt* inoculation. Equal amounts of RNA (15 μg) from different samples were resolved on denaturing 15% urea–polyacrylamide gels and transferred to Hybond‐N+ membranes (Amersham Biosciences, Mississauga, ON, Canada) using a semidry blotter (Bio‐Rad). Transferred RNA was then cross‐linked to the membrane by UV with an energy output of 1200 μJ/cm^2^ for 5 min. DIG‐labelled DNA probes were synthesized using target fungal gene sequences as template. Prehybridization was performed using DIG Easy Hybridization buffer (Sigma‐Aldrich) followed by hybridization at 42 °C overnight. The membranes were washed twice with 1× SSC, 0.1% SDS for a total of 15 min at 42 °C. Detection of the hybridized probe was performed using the DIG Nucleic Acid Detection Kit (Sigma‐Aldrich) following the manufacturer instructions. Photoemissions were captured using a ChemiDoc‐IT Imaging System (Bio‐Rad).

## Supporting information


**Figure S1** Molecular analysis of transgenic wheat T_1_ lines. Integration of hp‐*PtMAPK1*RNAi or hp‐*PtCYC1*RNAi construct in transgenic plants analyzed by PCR‐amplification of the corresponding *PtMAPK1* (a) or *PtCYC1* (b) transgenes. Genomic DNA extracted from transgenic and non‐transformed control (NTC) plants prior to *Pt* inoculation was used as template in PCR reactions. Expression of *PtMAPK1*RNAi or hp‐*PtCYC1*RNAi in the same plants analysed by RT‐PCR using primers specific to the corresponding *PtMAPK1* (c) or *PtCYC1* (d) transgene. cDNA prepared from total RNA extracted from transgenic and control plants prior to *Pt* inoculations was used as template in RT‐PCR reactions. M indicates 1 kb DNA ladder (Invitrogen); plasmid DNA served as positive control (PC). The PCR products were fractionated on a 1% agarose gel. Results from representative plants of each selected line are shown (lanes 1–20).

**Figure S2** Delay in progression of symptoms in leaves of hp‐*PtMAPK1*RNAi or hp‐*PtCYC1*RNAi expressing wheat T_1_ plants. *Pt* inoculated transgenic leaves are characterized by a slower and more restrictive uredinia development as compared to non‐transformed control Fielder plants. Representative leaves from transgenic T_1_ lines MAPK1‐2166 and CYC1‐2236 are shown. Photographs were taken at 6 dpi.

**Figure S3** Molecular analysis of transgenic wheat T_2_ lines. Integration of hp‐*PtMAPK1*RNAi or hp‐*PtCYC1*RNAi constructs in transgenic plants analyzed by PCR‐amplification of the corresponding *PtMAPK1* (a) and *PtCYC1* (b) transgenes. Genomic DNA extracted from transgenic and non‐transformed control (NTC) plants prior to *Pt* inoculation was used as template in PCR reactions. Expression of *PtMAPK1*RNAi or hp‐*PtCYC1*RNAi in the same plants was analysed by RT‐PCR using primers specific to the corresponding *PtMAPK1* (c) or *PtCYC1* (d) transgene. cDNA prepared from total RNA extracted from transgenic and control plants prior to *Pt* inoculations was used as template in RT‐PCR reactions. M indicates 1 kb DNA ladder (Invitrogen); BC, buffer control; plasmid DNA served as positive control (PC). The PCR products were fractionated on a 1% agarose gel. Results from representative plants of each selected line are shown (lanes 1–3).

**Figure S4** Delay in progression of symptoms in leaves of hp‐*PtMAPK1*RNAi or hp‐*PtCYC1*RNAi expressing wheat T_2_ plants. *Pt* inoculated transgenic leaves are characterized by a slower and more restrictive uredinia development as compared to non‐transformed control Fielder plants (NTC). Representative leaves from transgenic T_2_ lines MAPK1‐2166 and CYC1‐2236 are shown. Photographs were taken at 6 dpi.

**Table S1** Primers used in the research.
**Table S2** Segregation analysis of transgenic wheat T_1_ lines.
