# Supplementary figures and images for: Host‐induced silencing of essential genes in Puccinia triticina through transgenic expression of RNAi sequences reduces severity of leaf rust infection in wheat
Source: Plant Biotechnol J. 2017 Dec 15;16(5):1013–23. doi: 10.1111/pbi.12845 (PMC5902777; doi:10.1111/pbi.12845)

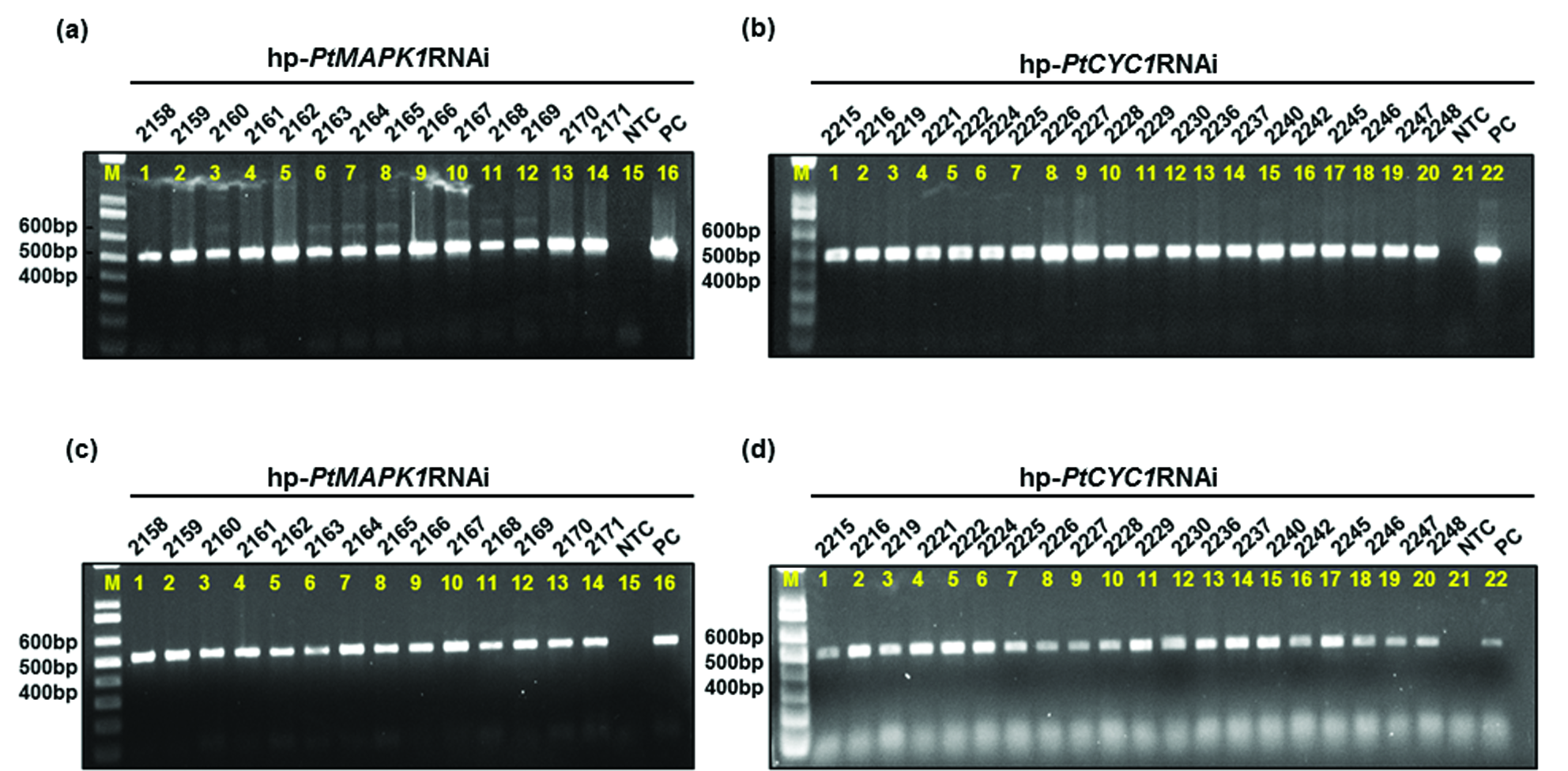

Supplement: Supplementary file 1 — Figure S1 Molecular analysis of transgenic wheat T1 lines. Integration of hp‐PtMAPK1RNAi or hp‐PtCYC1RNAi construct in transgenic plants analyzed by PCR‐amplification of the corresponding PtMAPK1 (a) or PtCYC1 (b) transgenes. Genomic DNA extracted from transgenic and non‐transformed control (NTC) plants prior to Pt inoculation was used as template in PCR reactions. Expression of PtMAPK1RNAi or hp‐PtCYC1RNAi in the same plants analysed by RT‐PCR using primers specific to the corresponding PtMAPK1 (c) or PtCYC1 (d) transgene. cDNA prepared from total RNA extracted from transgenic and control plants prior to Pt inoculations was used as template in RT‐PCR reactions. M indicates 1 kb DNA ladder (Invitrogen); plasmid DNA served as positive control (PC). The PCR products were fractionated on a 1% agarose gel. Results from representative plants of each selected line are shown (lanes 1–20). [file PBI-16-1013-s004.tif]

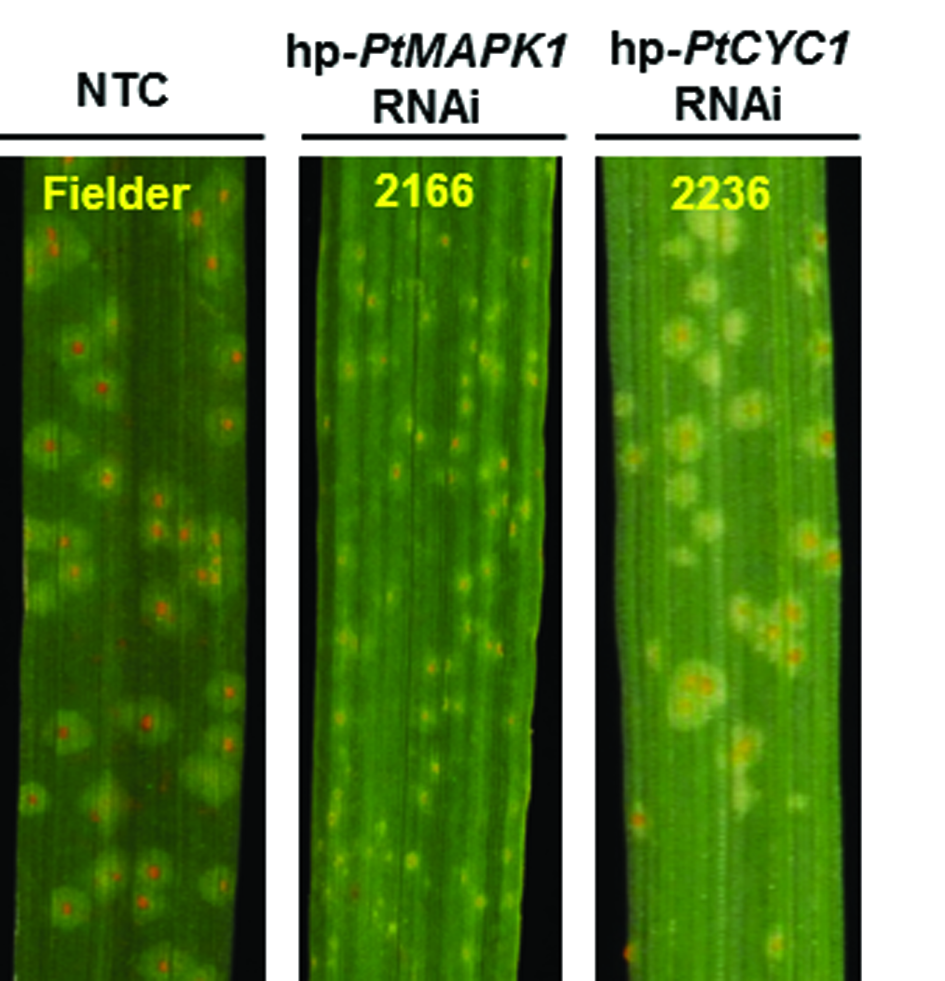

Supplement: Supplementary file 2 — Figure S2 Delay in progression of symptoms in leaves of hp‐PtMAPK1RNAi or hp‐PtCYC1RNAi expressing wheat T1 plants. Pt inoculated transgenic leaves are characterized by a slower and more restrictive uredinia development as compared to non‐transformed control Fielder plants. Representative leaves from transgenic T1 lines MAPK1‐2166 and CYC1‐2236 are shown. Photographs were taken at 6 dpi. [file PBI-16-1013-s003.tif]

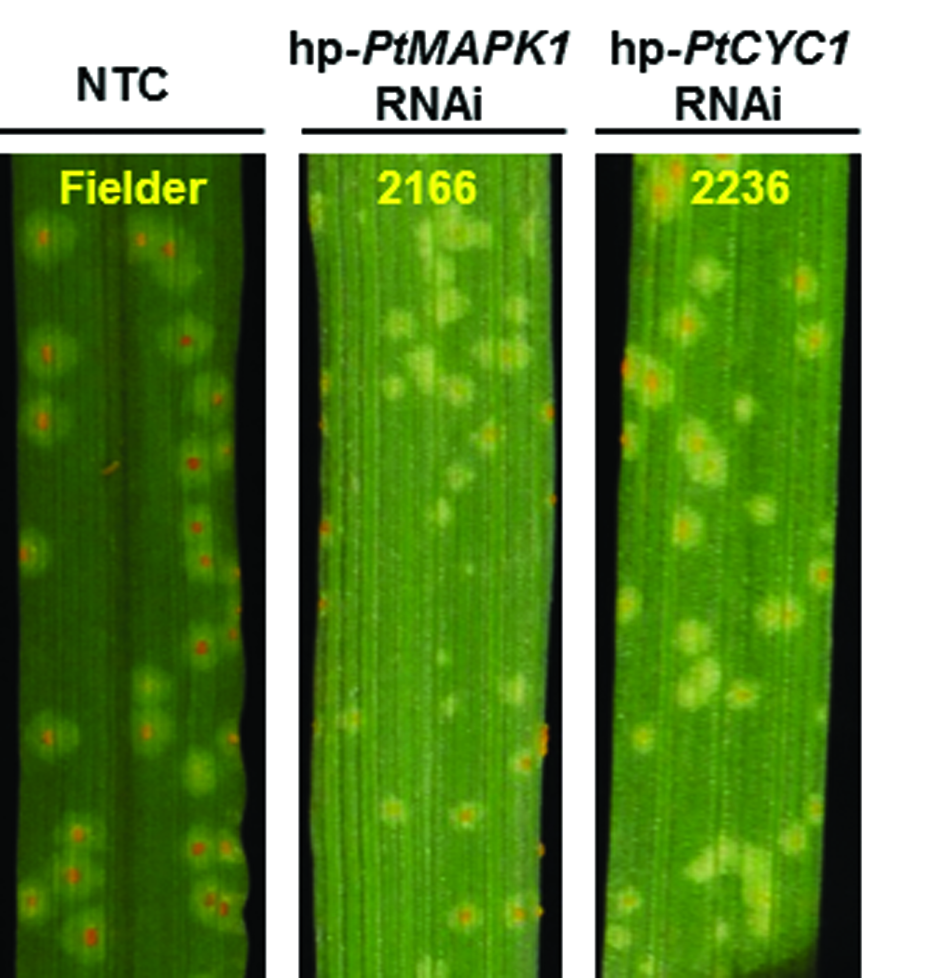

Supplement: Supplementary file 4 — Figure S4 Delay in progression of symptoms in leaves of hp‐PtMAPK1RNAi or hp‐PtCYC1RNAi expressing wheat T2 plants. Pt inoculated transgenic leaves are characterized by a slower and more restrictive uredinia development as compared to non‐transformed control Fielder plants (NTC). Representative leaves from transgenic T2 lines MAPK1‐2166 and CYC1‐2236 are shown. Photographs were taken at 6 dpi. [file PBI-16-1013-s002.tif]
