# Supplementary material for: Host‐induced silencing of essential genes in Puccinia triticina through transgenic expression of RNAi sequences reduces severity of leaf rust infection in wheat
Source: Plant Biotechnol J. 2017 Dec 15;16(5):1013–23. doi: 10.1111/pbi.12845 (PMC5902777; doi:10.1111/pbi.12845)
Supplement: Supplementary file 5 — Table S1 Primers used in the research. Table S2 Segregation analysis of transgenic wheat T1 lines. [file PBI-16-1013-s001.docx]

**Supporting information**

**Table S1.** Primers used in the research

| **Primer** | **Sequence (5'-3')** |
| --- | --- |
| **HD-RNAi vector construction** | |
| *PtMAPK1*-F | CACCAGATCTCCGATGGCACAGAGCCCCCGAG |
| *PtMAPK1*-R | TCAAGTGGTAAAGTGAAAGCTG |
| *PtCYC1*-F | TTACTAGTGGCGCGCCATGTCAGAAAACCAAAACTGCTA |
| *PtCYC1*-R | TTGCGATCGCCCTAGGTCAGAGGGTGCCACAGTCAGCG |
| **qPCR analysis** | |
| *PtMAPK1*-qPCR-F | TTGAAGCCATCGAACCTTTT |
| *PtMAPK1*-qPCR-R | TGGCTTTGGTGTATTGCTTG |
| *PtCYC1*-qPCR-F | GGTGAAGTTGTCAAGGGCTTC |
| *PtCYC1*-qPCR-R | GTATGACTCGCTTCTGGATGG |
| Ta-EF1-qPCR-F | GGTGATGCTGGCATAGTGAA |
| Ta-EF1-qPCR-R | GATGACACCAACAGCCACAG |
| *Pt*-RTP1-qPCR-F | CGGAAGAATAGCCGGAAAATG |
| *Pt*-RTP1-qPCR-R | CTTAGACATCTCGATGTCTCG |
| *Pt*-SDH-qPCR-F | GGTTCCAGCGATAGATCGAG |
| *Pt*-SDH-qPCR-R | CAACTACGACCAGCCACTCA |

**Table S2.** Segregation analysis of transgenic wheat T_1_ lines

| **HD-RNAi constructs and line number** | **T_1_ generation^a^** | | **Segregation** | **χ^2^** | ***P*^b^** |
| --- | --- | --- | --- | --- | --- |
|  | **Positive** | **Negative** | **ratio tested** |  |  |
| **hp-*PtMAPK1*RNAi** |  |  |  |  |  |
| MAPK1-2159 | 15 | 6 | 3:1 | 0.14 | 0.70 |
| MAPK1-2162 | 12 | 5 | 3:1 | 0.17 | 0.67 |
| MAPK1-2163 | 12 | 6 | 3:1 | 0.66 | 0.41 |
| MAPK1-2166 | 23 | 6 | 3:1 | 0.28 | 0.59 |
| MAPK1-2169 | 18 | 7 | 3:1 | 0.12 | 0.72 |
| MAPK1-2170 | 16 | 7 | 3:1 | 0.36 | 0.54 |
| **hp-*PtCYC1*RNAi** |  |  |  |  |  |
| CYC1-2216 | 14 | 4 | 3:1 | 0.07 | 0.78 |
| CYC1-2224 | 15 | 4 | 3:1 | 0.15 | 0.69 |
| CYC1-2226 | 6 | 3 | 3:1 | 0.33 | 0.56 |
| CYC1-2227 | 11 | 1 | 15:1 | 0.08 | 0.76 |
| CYC1-2228 | 14 | 6 | 3:1 | 0.26 | 0.60 |
| CYC1-2236 | 15 | 6 | 3:1 | 0.14 | 0.70 |
| CYC1-2248 | 13 | 5 | 3:1 | 0.07 | 0.78 |

^a^Data are based on PCR analysis for detection of respective *PtMAPK1* and *PtCYC1* transgene.

^b^Probability that observed ratios reflect expected segregation ratio of 3:1 or 15:1.
